# Editorial for the Special Issue: “Fungal Pathogenicity Factors”

**DOI:** 10.3390/pathogens12040539

**Published:** 2023-03-30

**Authors:** Xin Guan, Flora Pensec

**Affiliations:** 1 College of Horticulture and Landscape Architecture, Southwest University, Chongqing 400715, China; xin.guan@swu.edu.cn; 2 Key Laboratory of Agricultural Biosafety and Green Production of Upper Yangtze River, Ministry of Education, Chongqing 400715, China; 3 Laboratoire Universitaire de Biodiversité et Écologie Microbienne, University Brest, INRAE, F-29280 Plouzané, France

Pathogenicity factors are important aspects of the arsenal of fungal agents, allowing them to infect a broad range of hosts or to specifically target a crop by being capable of evading host defenses or having enzymatic activities that target plant tissues. Research programs concerning these pathogenicity factors allow us to gain insight into infection mechanisms, and thus, help us better orientate the selection programs of resistant plants.

The current Special Issue, entitled “Fungal Pathogenicity Factors”, is composed of six original articles and one review and aims to characterize different aspects of fungi pathogenicity factors and their capacity to induce plant defense responses.

In this Special Issue, four publications focus on the fungus side in order to decipher their pathogenicity factors. In their review, Belair et al. [1] highlight the knowledge gained over this past decade on genes encoding effectors, carbohydrate-associated enzymes (CAZyme), transporters and genes associated with secondary metabolism, their representativeness within the Botryosphaeriaceae family, and their expression during grapevine infection. They indicate that further functional studies are needed in order to better elucidate the pathogenicity mechanisms of this family. In their original article, Boufleur et al. [2] focus on a specific class of factors: effectors, described as small secreted proteins that have no homology to any other protein or that have homology to proteins from the same genus or species. Their study enables us to compare the effector repertoire of different *Colletotrichum* species infecting soybeans, and highlight the fact that species from different lineages do not share any candidate effectors, suggesting that these lineages acquired the ability to infect soybeans independently. The study of the effector repertoire enables us to have a broad view of the arsenal of a fungal species, and complementary functional analyses allow for the characterization of the role of a candidate effector. An effector identified as a metalloprotease by Zhang et al. [3] is described by both transcriptomic and functional analyses to highly contribute to *Fusarium oxysporum* f. sp. *cubense* pathogenicity on banana plants by inhibiting host immunity. Infection kinetics are generally characterized by the up-regulation of genes encoding effectors at an early stage of the infection, and then genes encoding CAZymes are generally up-regulated and constitute the start of a necrotrophic phase. A specific CAZyme, glycosyltransferase, is analyzed by Zhang et al. [4]. This protein secreted by the necrotrophic fungi *Rhizoctonia solani* is described to be conserved in primary fungal taxonomic categories. Interestingly, this glycosyltransferase may be able to suppress cell death in addition to its enzymatic activity, allowing the pathogen to evade plant defense mechanisms such as H_2_O_2_ production. 

The above studies in this current Special Issue have reported the induction of defense responses in different plant tissues after natural or artificial inoculation with phytopathogenic pathogens. Despite the activation of plant defense mechanisms, it seems that many of the pathogens can finally conquer plant obstacles and efficiently colonize plant tissues. In the frame of the plant–pathogen classic zigzag model, such successful pathogenic pathogens have screened out a class of sensitive plant species. On the contrary, there is a class of resistant plant species which can repress the invasion of pathogens. From the view of plants, this plant–pathogen battle covers initial pathogen-triggered innate immunity and pathogenic effector-triggered innate immunity genetically and morphologically. In this context, the invasion of pathogens from the epidermis of plant organs to the vascular tissues rouses the cumulative progression of plant responses. This can be either a systemic spread of fungal spores and phytotoxins mainly restricted to the vessel lumen and cells surrounding vessels (e.g., plant vascular wilt disease), or the type that hardly spreads systemically due to the death of a portion of vascular cambium that disables the newly formed functional xylem and phloem (e.g., canker) (Pouzoulet et al., 2014) [5]. However, what if the plant living cells stop working? Therefore, the early fungi development process matters. Before the formation of embolism/occlusion of vessels or the cell death of cambium, there must be responses from living cells. 

The contribution to the current Special Issue from Belair et al. [1] reviews the cell wall thickening in paratracheal parenchyma that impedes lateral hyphae penetration in xylem parenchyma cells and looks at cell wall modifications in living fibers and ray parenchyma that are associated with suberin rather than lignin deposits. The contribution by Sun et al. [6] to the current Special Issue identifies 15 genes encoding stress-associated proteins (SAPs) from grapevines (*Vitis vinifera*) via sequence comparisons. The tissue- and development-specific expressions were analyzed with database help.

In recent decades, the ban of numerous phytosanitary products has limited the number of solutions to treat fungal diseases. The sustainable management of biological control has attracted considerable attention due to its advantages in the balance of crop production and agricultural environment protection. Among them, some Trichoderma strains can confer biocontrol either directly by interacting with pathogens via mycoparasitism, or via competition for nutrients or root niches, while other strains establish a robust and durable colonization of root surfaces and penetrate into the epidermal cells to indirectly induce the host resistance, thus enhancing root growth (Harman et al., 2004) [7]. The contribution by Xu et al. [8] to the current Special Issue addresses how maize–soybean relay strip intercropping significantly increases the density and composition proportion of beneficial Trichoderma to antagonize the pathogenic Fusarium species in the rhizosphere, thus potentially contributing to the suppression of soybean root rot under the intercropping. Moreover, B. velezensis is identified as a plant growth-promoting biocontrol agent with the ability to produce IAA and cytokinin and solubilize mineral phosphate (Lugtenberg et al., 2009) [9]. The contribution by Yin et al. [10] to the current Special Issue reports a B. velezensis strain GSBZ09 isolated from the rhizosphere soil of grapevines. It has antagonistic activities against a broad range of fungal and bacterial pathogens in grapevines. The biocontrol effect on grapevine white rot, the antifungal mechanism, and the plant growth-promoting ability were evaluated both in vitro and in vivo.

All seven contributions to the current Special Issue cover a wide range of topics focusing on fungi pathogenic factors and on their induction of plant defense responses. It is important to attain a molecular understanding of a phenomenon through physiological and morphology approaches. The plant–pathogen interaction is an endless coevolution process. Understanding the mechanisms will facilitate the investigation of new control solutions and help in breeding new cultivars resistant to certain diseases.
